# Biallelic variants in *DNAH11* cause male infertility with asthenozoospermia in a Chinese non-consanguineous family: A case report

**DOI:** 10.1097/MD.0000000000046556

**Published:** 2025-12-12

**Authors:** Yan Zhang, Junlin Pan, Jinwei Hou, Na Shi, Huiling Qu, Longhuan Jiang, Haiping Liu

**Affiliations:** a Department of Reproductive Medicine, The 960th Hospital of the PLA Joint Logistics Support Force, Jinan, China.

**Keywords:** Asthenozoospermia, *DNAH11*, male infertility, primary infertility, whole-exome sequencing

## Abstract

**Rationale:**

Asthenozoospermia (AZS), a leading cause of male infertility, frequently results from genetic defects that disrupt flagellar assembly. Mutations in the *DNAH11* gene can cause abnormal dynein arm structures, leading to AZS and impaired male fertility. However, current evidence regarding *DNAH11* mutations in male infertility remains limited, warranting further exploration of their pathogenic mechanisms.

**Patient concerns:**

A 35-year-old Chinese male presented with primary infertility and AZS.

**Diagnoses:**

Whole-exome sequencing was performed on the proband to identify the potential genetic etiology, followed by Sanger sequencing validation of candidate variants in both the proband and his parents. A novel compound heterozygous mutation in the *DNAH11* gene – comprising a frameshift mutation (c.1174del) and a splicing mutation (c.3766-8A > G) – was identified in the proband. Sanger sequencing confirmed paternal inheritance of the c.1174del mutation and maternal inheritance of the c.3766-8A > G mutation.

**Interventions:**

After receiving genetic counseling, the patient chose to undergo intracytoplasmic sperm injection (ICSI) treatment.

**Outcomes:**

The ICSI procedure did not result in a successful clinical pregnancy. Follow-up outcomes will continue to be monitored.

**Lessons:**

This study identified novel biallelic *DNAH11* variants associated with reduced sperm motility, expanding the known mutational spectrum of *DNAH11* variants implicated in AZS and providing additional evidence for genetic counseling and diagnosis.

## 1. Introduction

Infertility is a major global health concern, affecting approximately 17.5% of couples attempting to conceive, with nearly 50% of cases attributed to male factors.^[1,2]^ Male infertility is a multifactorial disorder with a substantial genetic basis and highly heterogeneous clinical manifestations. Asthenozoospermia (AZS) is one of the most prevalent male infertility factors, characterized by decreased sperm motility and diagnostically defined by either subthreshold progressive motility (<32%) or total motility (<40%).^[3]^ Spermatogenesis is a highly complex process regulated by the interaction of multiple genetic and environmental factors.^[4]^ Although genetic causes have been identified in a subset of patients with AZS, the underlying pathogenic mechanisms remain largely unclear in most affected individuals.

Primary ciliary dyskinesia (PCD) is a rare genetic disorder characterized by dysfunctional cilia, which can lead to multisystem complications.^[5]^ Because sperm flagella are specialized ciliary structures, PCD patients often exhibit impaired sperm motility that results in infertility.^[6,7]^ Although the molecular mechanisms of PCD in respiratory diseases have been well elucidated, research on its pathogenic mechanisms in male infertility remains relatively limited. To date, more than 40 genes associated with PCD have been identified as significantly related to male infertility, including *CCDC39/40, DNAAF2/4/6*, and *DNAH2/6/17*, among others.^[8–10]^

*DNAH11* (dynein axonemal heavy chain 11, OMIM: 603339) is one of the most frequently implicated pathogenic genes in PCD. It encodes a dynein protein that is essential for ciliary motility.^[11]^ Mutations in the *DNAH11* gene can cause structural abnormalities in the dynein arm, leading to AZS and impaired male fertility.^[12,13]^ However, data on *DNAH11*-related pathogenic variants in male infertility remain limited. Therefore, additional genetic studies are needed to further elucidate the pathogenic mechanisms of *DNAH11* in male infertility.

With the advancement of sequencing technologies, high-throughput sequencing has become a standard diagnostic approach for identifying the etiological causes of AZS in clinical practice.^[14,15]^ The objective of the present study was to characterize the molecular basis of *DNAH11* variants in a male patient with AZS. We identified a male infertility patient with AZS carrying a compound heterozygous mutation in the *DNAH11* gene. This study provides new genetic evidence supporting the causal relationship between *DNAH11* mutations and male infertility, thereby contributing to the improved diagnosis and genetic counseling of affected individuals.

## 2. Case presentation

### 2.1. Patients

This study enrolled a 35-year-old man with primary infertility from the Department of Reproductive Medicine at The 960th Hospital of the People’s Liberation Army (PLA) Joint Logistics Support Force. The patient’s parents were non-consanguineous and had no notable family history of genetic disorders. The patient exhibited normal bilateral testes, epididymides, and vas deferens, with hormone levels within normal ranges. His karyotype was 46, XY, and no Y chromosome microdeletions were detected. The patient’s wife had regular menstruation, normal sex hormone levels, normal thyroid function, a normal ovarian reserve, and a normal karyotype (46, XX). Neither spouse had a history of exposure to reproductive toxicants. Routine semen analysis revealed AZS in this patient. Multiple semen analyses confirmed the diagnosis, showing consistently low sperm progressive motility values below the reference threshold of 32%. General information about the patient with primary infertility is presented in Table 1. This study was approved by the Ethics Committee of The 960th Hospital of the People’s Liberation Army (PLA) Joint Logistics Support Force. After obtaining signed written informed consent, peripheral blood samples were collected from the patient and his parents for subsequent sequencing analyses.

**Table 1:** General information of the patient with *DNAH11* gene variants in this study.

Clinical characteristics	Value	Reference range
Age (year)	35	–
Duration of infertility (year)	5	–
Testicular size (left, mL)	12	–
Testicular size (right, mL)	12	–
Semen volume (mL)	3	>1.5
Semen concentration	8.15	>15.0
Semen pH	7.2	–
The total number of sperm n (10^6^ per mL)	24.44	>39.0
Progressive motility (%)	11.76	>32.0
Total motility (PR + NP, %)	15.2	>40.0
DNA fragmentation index (DFI, %)	18.49	–
High DNA stainability (HDS, %)	13.12	–
Follicle-stimulating hormone (FSH, mIU/mL)	4.23	0.9–10.9
Luteinizing hormone (LH, mIU/mL)	2.16	2.8–6.8
Prolactin (PRL, ng/mL)	9.47	4.1–18.49
Estradiol (E2, pg/mL)	31	16–44.5
Testosterone (T, ng/mL)	4.16	1.2–10.19
Karyotype	46, XY	–
Y chromosome microdeletions	No	–
Clinical diagnosis	Asthenozoospermia	–
PCD-related phenomenon	No	–

DNAH11 = dynein axonemal heavy chain 11, PCD = primary ciliary dyskinesia.

### 2.2. Whole-exome sequencing

Genomic DNA was extracted from 2 mL of whole blood using the QIAamp DNA Blood Mini Kit (Qiagen, Germany). Targeted enrichment of genomic regions was performed using the Agilent SureSelect Human Exon Capture Kit (Agilent, USA) through multiple probe hybridizations. The captured products were purified with Agencourt AMPure XP beads (Beckman Coulter, USA). The purified DNA was then processed using the TruePrep™ DNA Library Prep Kit V2 for Illumina (Vazyme, China), and unique indices were incorporated withusing the TruePrep™ Index Kit V2 for Illumina (Vazyme, China). Library quality was assessed with a Qubit Fluorometer and an Agilent High Sensitivity DNA Kit (Agilent, USA). Quantification was performed using the Illumina DNA Standards and Primer Premix Kit (Kapa Biosystems, USA). Finally, the prepared libraries were subjected to massively parallel sequencing on the Illumina HiSeq 2500 platform (Illumina, USA).

### 2.3. Data analysis

Filtered sequencing reads were aligned to the human reference genome (hg19) using the Burrows–Wheeler Aligner. Variant calling was performed using the Genome Analysis Toolkit, and variant annotation was carried out with ANNOVAR, referencing public databases such as the Database of Single Nucleotide Polymorphisms, the 1000 Genomes Project, and the Exome Aggregation Consortium. Variants with a minor allele frequency >5% were excluded, except those classified as pathogenic or likely pathogenic in the Human Gene Mutation Database (HGMD) or ClinVar. Functional impact predictions were evaluated using several in silico tools, including SIFT, PolyPhen-2, mutation taster, and REVEL. Candidate variants were prioritized according to clinical relevance and classified following the guidelines of the American College of Medical Genetics and Genomics (ACMG).

### 2.4. Sanger sequencing

Candidate variants were validated by Sanger sequencing using variant-specific primers. polymerase chain reaction amplification was performed according to the manufacturer’s instructions for the TIANamp Universal DNA Purification Kit (TIANGEN, China). The amplified polymerase chain reaction products were purified and subsequently sequenced on an ABI 3730xl DNA Analyzer (Thermo Fisher Scientific, USA).

### 2.5. Identification of DNAH11 compound heterozygous mutations

**Figure 1.**
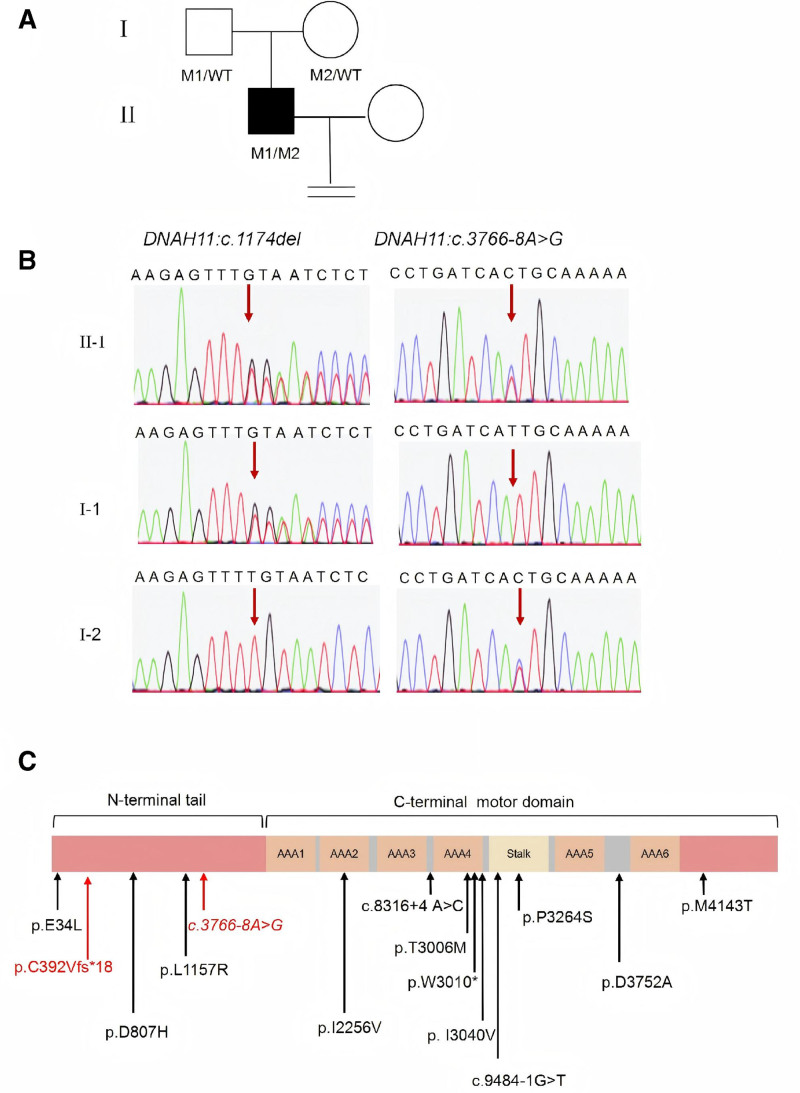
Genetic information of a male infertility family. (A) Pedigree of a male infertility family. (B) Consequence of Sanger sequencing. (C) Summary of *DNAH11* mutations specific positions of previously reported in male infertility patients together with the identified mutation in this study. The mutation reported in this study is highlighted in red. DNAH11 = dynein axonemal heavy chain 11

To elucidate the genetic basis of the patient’s AZS, whole-exome sequencing (WES) was performed. Bioinformatic analysis identified 2 heterozygous mutations – c.1174del (p.C392Vfs*18) and c.3766-8A > G – in the *DNAH11* gene (Fig. 1A). Sanger sequencing validated these 2 variants in the family and demonstrated that the c.1174del mutation was inherited from the father, whereas the c.3766-8A > G variant was inherited from the mother (Fig. 1B). The c.1174del mutation has not been previously reported in either the ClinVar or the HGMD, suggesting that it represents a novel variant. Although the c.3766-8A >G variant is classified as likely pathogenic in the ClinVar database, it has not yet been recorded in HGMD. The c.1174del frameshift variant, located in exon 6, introduces a premature termination codon that produces a truncated protein containing 17 incorrectly encoded amino acids. This truncated protein retains 391 correctly encoded amino acids forming part of the N-terminal tail. However, the c.1174del mutation is predicted to eliminate the AAA (ATPases Associated with diverse cellular Activities) 1 to 6 domains and the stalk-like structure between AAA4 and AAA5, leading to a loss of protein function. Bioinformatic analysis using splicing prediction tools (SpliceAI: 0.99, MaxEntScan: 4.83, dbscSNV1_ADA: 1.0, dbscSNV1_RF: 0.95) indicated that the c.3766-8A > G variant is likely to affect mRNA splicing. According to the ACMG guidelines, the c.1174del variant was classified as likely pathogenic (PVS1 + PM2_Supporting), whereas the c.3766-8A > G variant was categorized as of uncertain significance (PM3 + PM2_Supporting + PP3).

Intracytoplasmic sperm injection (ICSI) is an established therapeutic approach for treating male factor infertility. To evaluate potential treatment strategies for *DNAH11* variant–associated male infertility, ICSI was performed for the patient harboring the *DNAH11* mutation. After comprehensive genetic counseling, the patient consented to undergo ICSI treatment. Unfortunately, the procedure did not result in a successful clinical pregnancy. Follow-up evaluations are ongoing.

A systematic search of the PubMed database using the keywords “DNAH11” and “male infertility” identified 9 cases from 8 independent pedigrees, involving 12 distinct *DNAH11* gene mutations (Fig. 1C). As summarized in Supplementary Table 1, Supplemental Digital Content, https://links.lww.com/MD/Q914, we conducted a comprehensive analysis of the clinical phenotypes and genotypes of these patients. Notably, only 1 patient exhibited mild PCD-related symptoms, including chronic cough and bronchiectasis, whereas the remaining 8 patients showed no typical PCD manifestations. This observation suggests that *DNAH11* mutations can cause isolated male infertility without the characteristic PCD-associated symptoms.

## 3. Discussion

In this study, we identified a biallelic variation in the *DNAH11* gene in a patient with male infertility due to AZS. The biallelic *DNAH11* variants were associated with AZS, but no PCD-related phenotype was observed. This finding expands the known spectrum of pathogenic *DNAH11* variants associated with male infertility and underscores the importance of genetic testing in diagnosing infertility-related pathogenic variants, particularly those involving *DNAH11*.

Substantial evidence indicates that *DNAH11* mutations represent a major molecular cause of PCD, given the gene’s essential role in regulating respiratory ciliary motility.^[16]^ Because sperm flagella are specialized forms of motile cilia, PCD patients frequently exhibit impaired sperm motility leading to infertility.^[17,18]^ However, few studies have investigated whether *DNAH11* variants directly contribute to male infertility through defects in sperm motility. In 2008, Zuccarello et al first identified 3 of 90 patients with AZS carrying heterozygous *DNAH11* mutations.^[19]^ Subsequently, Zhu et al screened *DNAH11* in 87 Chinese patients with idiopathic asthenozoospermia and identified 1 individual harboring compound heterozygous *DNAH11* mutations (c.9484-1G > T/c.12428T > C).^[20]^ Later, Guo et al analyzed WES data from 975 male infertility patients and identified 7 novel *DNAH11* mutations in 4 patients with asthenoteratozoospermia.^[21]^ Long, et al reported WES data of 38 male infertility patients and found 2 mutations in DNAH11 in 1 patients with asthenoteratozoosperm.^[14]^ In the present study, we also identified a male infertility patient with AZS carrying compound heterozygous *DNAH11* mutations (c.1174del/c.3766-8A > G). Combining the current findings with previous reports, among ten male infertility patients carrying *DNAH11* mutations, 9 exhibited no clinical manifestations of PCD, while only one presented with mild PCD-related symptoms, including chronic cough and bronchiectasis. Based on this evidence, we propose that *DNAH11* mutations demonstrate notable phenotypic heterogeneity, with some carriers developing PCD and others exhibiting only male infertility. This observation suggests possible mutation-specific effects or tissue-selective pathogenic mechanisms underlying *DNAH11*-associated disorders.

The DNAH11 protein comprises an N-terminal region, 6 AAA domains (ATPases Associated with diverse Cellular Activities), a microtubule-binding domain, 2 coiled-coil segments, and a C-terminal region. In this study, the identified compound heterozygous *DNAH11* mutations (c.1174del and c.3766-8A > G) were both located within the N-terminal region. The c.1174del frameshift mutation in exon 6 introduces a premature stop codon, producing a truncated protein that retains 391 N-terminal amino acids but lacks all AAA1 to 6 domains and the stalk-like structure between AAA4 and AAA5, resulting in a complete loss of protein function. To our knowledge, the c.1174del mutation in the *DNAH11* gene is first reported in this study. This mutation has not been previously documented or listed in the Genome Aggregation Database. According to the ACMG guidelines, this mutation was classified as likely pathogenic, and its pathogenic mechanism may involve abnormal transcript generation and nonsense-mediated mRNA decay. Although the c.3766-8A > G variant was classified as likely pathogenic in the ClinVar database, it has not been reported in the literature or the HGMD. Furthermore, bioinformatic analysis using multiple splicing prediction tools consistently predicted that the c.3766-8A > G variant in *DNAH11* would significantly disrupt normal mRNA splicing. Following the ACMG guidelines, the c.3766-8A > G variant was categorized as of uncertain significance, and its potential role in male infertility remains unclear, warranting further investigation.

Sperm quality (including count, motility, and morphology) is a determining factor for male fertility. Extensive literature indicates that AZS significantly reduces the success rates of both natural conception and assisted reproductive technology.^[22–24]^ In this study, the patient’s semen analysis met the diagnostic criteria for AZS, as both the sperm concentration and the percentage of progressively motile sperm were below the World Health Organization reference limits. This finding provides an etiological explanation for the patient’s prolonged infertility, identifying AZS as the root cause. This is consistent with existing literature, which demonstrates that severe abnormalities in semen parameters impair male reproductive potential.

Although this study provides valuable evidence suggesting a potential association between *DNAH11* variants and AZS, several limitations should be acknowledged. First, the sample size is relatively small, as only a single case was analyzed, emphasizing the need to expand the patient cohort to validate these findings. Second, elucidating the precise pathogenic mechanism by which *DNAH11* mutations lead to AZS requires additional experimental investigations. Future studies will focus on in-depth mechanistic analyses to further clarify the pathogenic pathways linking *DNAH11* mutations to male infertility.

## 4. Conclusion

In summary, the compound heterozygous *DNAH11* mutations c.1174del and c.3766-8A > G may represent the pathogenic cause of AZS in this male infertility patient. These findings expand the known mutational spectrum of the *DNAH11* gene and provide valuable additional information for genetic counseling in this family. Collectively, these results establish an evidence-based foundation for incorporating genetic testing into counseling and therapeutic decision-making for AZS-associated male infertility.

## Acknowledgments

We thank the patient and his family members for participating in this study and sharing their stories.

## Author contributions

**Conceptualization:** Haiping Liu.

**Data curation:** Junlin Pan, Jinwei Hou.

**Investigation:** Na Shi.

**Methodology:** Huiling Qu, Longhuan Jiang.

**Writing – original draft:** Yan Zhang.

**Writing – review & editing:** Yan Yang, Junlin Pan, Jinwei Hou, Na Shi, Huiling Qu, Longhuan Jiang, Haiping Liu.

## Supplementary Material
