# Supplementary material for: Biallelic variants in DNAH11 cause male infertility with asthenozoospermia in a Chinese non-consanguineous family: A case report
Source: Medicine (Baltimore). 2025 Dec 12;104(50):e46556. doi: 10.1097/MD.0000000000046556 (PMC12708164; doi:10.1097/MD.0000000000046556)
Supplement: Supplementary file 1 [file medi-104-e46556-s001.docx]

**Supplementary Table 1 Clinical phenotypes and genotypes of the 9 reported patients with *DNAH11* mutations**

| Clinical characteristics | Case 1 | Case 2 | Case 3 | Case 4 | Case 5 | Case 6 | Case 7 | Case 8 | Case 9 |
| --- | --- | --- | --- | --- | --- | --- | --- | --- | --- |
| Age (years) | 34 | 40 | 34 | 44 | 31 | 34 | 35 | 32 | NA |
| Duration of infertility (years) | NA | NA | NA | 12 | NA | NA | NA | NA | NA |
| Testicular size (left, ml) | NA | NA | NA | 15 | NA | NA | NA | NA | NA |
| Testicular size (right, ml) | NA | NA | NA | 15 | BA | NA | NA | NA | NA |
| Semen volume (ml) | NA | NA | NA | NA | 4.8 | 4.0 | 2.2 | 3.5 | NA |
| Semen concentration | NA | NA | NA | 1.28 | 7.5 | 14.1 | 9.8 | 6.4 | NA |
| The total number of sperm n (10^6^ per ml) | 23 | 36 | 21 | NA | 36.0 | 56.4 | 21.6 | 22.4 | NA |
| Progressive motility (%) | NA | NA | NA | 5.26 | 28.9 | 4.5 | 0 | 5.0 | NA |
| Total motility (PR + NP, %) | 2 | 14 | 10 | 21.5 | NA | NA | NA | NA | NA |
| Clinical diagnosis | Asthenozoospermia | Asthenozoospermia | Asthenozoospermia | Asthenozoospermia | Asthenoteratozoospermia | Asthenoteratozoospermia | Asthenoteratozoospermia | Asthenoteratozoospermia | Asthenoteratozoospermia |
| PCD-related phenomenon | NA | NA | NA | NA | Wet cough,  Bronchiectasis | NA | NA | NA | NA |
| *DNAH1*1 gene variants | c.9118 A>G(I3040V) | c.9118 A>G(I3040V) | c.9118 A>G(I3040V) | c.9484-1G>T, c.12428T>C(p.M4143T) | c.9029G>A  (p.W3010*) | c.3470T>G (p.L1157R  )/c.9790 C>T  (p.P3264) | c.100_101delinsTT  (p.E34L)/c.6766 A>G (p.I2256V) | c.2419G>C (p.D807H)/c.8316+4 A>C | c.9017C>T (p.T3006M)  c.11255A>C (p.D3752A) |
